# Quality of Life and Audiological Benefits in Pediatric Cochlear Implant Users in Romania: Systematic Review and Cohort Study

**DOI:** 10.3390/jpm13111610

**Published:** 2023-11-15

**Authors:** Gina Gundacker, Delia Emilia Trales, Horatiu Eugen Stefanescu

**Affiliations:** 1 Life Science Department, University of Applied Sciences Technikum Vienna, 1200 Vienna, Austria; 2 Department of Otolaryngology, Victor Babeș Medical and Pharmaceutical University, 300041 Timișoara, Romania; delia_trales@yahoo.com (D.E.T.); stefanescu@umft.ro (H.E.S.)

**Keywords:** quality of life, sensorineural hearing loss, cochlear implant, audiometry, rehabilitation

## Abstract

Profound sensorineural hearing loss (SNHL) can be successfully treated with a cochlear implant (CI), and treatment is usually accompanied by increased quality of life (QoL). Therefore, the aim of this study was to investigate generic and health-related QoL, as well as the level of audiological outcomes, of CI users, in addition to whether Qol can be restored to the extent of those with normal hearing. Furthermore, different implantation timepoints were compared (early vs. late), and a possible correlation between health and generic QoL questionnaires was investigated. The outcomes from 93 pediatric CI users from Romania were analyzed in the study. Two QoL questionnaires (SSQ12, AQoL-6D), as well as the HSM sentence test and Soundfield measurements, were assessed. The outcomes revealed that the CI users were able to achieve the same QoL as their age- and-gender matched peers with normal hearing, and hearing was restored with good speech comprehension. No significant difference between early- and late-implanted children was detected, although a tendency of a better Word Recognition Score (+10%) in the early-implanted group was discovered. A moderate and significant correlation between the generic and health-related Qol questionnaire was observed. Audiological examinations are still the standard practice by which to measure the benefit of any hearing intervention; nonetheless, generic and health-related QoL should be assessed in order to provide a full picture of a successful and patient-satisfactory cochlear implant procedure.

## 1. Introduction

Hearing loss (HL) is the most common sensory impairment, and, as of today, the only one which can be replaced with an implant [1]. According to the world report on hearing from 2021, conducted by the World Health Organization (WHO), more than 5% of the world’s population, i.e., roughly 460 million individuals, have moderate to severe hearing loss which would influence their everyday lives if left untreated [2]. Of these people, about 34 million children are affected with disabling hearing loss [3]. Hearing-impaired people suffer from restrictions regarding their participation in daily life [3]. Furthermore, individuals with communication difficulties have a risk of restricted cognitive skills, including reduced attention and concentration spans as well as lower than average memory recall [2]. This can be avoided if they receive the right support and treatment as soon as possible. Disabling hearing loss often also leads to low self-esteem, feelings of seclusion, and social isolation, causing depression and anxiety [2,3]. Hence, people who are hearing-impaired often report a lower overall quality of life (QoL) [2].

Depending on the type and severity of hearing impairment, several treatment options are possible, from conventional hearing aids to medical interventions utilizing passive middle ear prostheses, active middle ear or bone conduction implants, and cochlear implants (CI). Cochlear implantation often requires personalized rehabilitation to not only hear sounds, but to enhance speech perception, speech production, and language development [3,4]. With the use of a CI, approximately one million partly or completely deaf individuals worldwide have been able to regain functional hearing [5]. The literature on children with CIs has demonstrated significant outcomes, especially in the development and perception of speech [6]. Children who have received cochlear implants at a young age have developed speech perception skills that allow them to develop fluent spoken language at a rate resembling that of children with normal hearing [6,7].

The progress and benefit of a cochlear implant can be measured via objective measures such as pure tone audiometry, which is measured at certain frequencies and sound levels [8]. Also, sentence tests are regularly applied in order to check whether speech comprehension is developing. The Hochmair–Schulz–Moser (HSM) sentence test is one example of such an assessment, and was developed especially for CI users, applying everyday words to test their speech perception [9]. In general, beneficial audiometric measurements do not always indicate a better QoL [10]. However, since the aim of such interventions is to improve quality of life, attention should also be focused on how the person subjectively perceives their new hearing. A vast amount of different QoL questionnaires, validated and translated into numerous languages, are available and are used to validate the benefit to the patient. These tests can be taken into account for future treatments and can be compared to other, similar interventions [11].

Quality of life questionnaires validate individuals’ subjective perception of different dimensions of life, such as mobility, mental health, sight, hearing, etc. One can distinguish between generic and disease-specific QoL questionnaires and their respective impacts on quality of life [10]. The evaluation of quality of life is still not a standard clinical routine measure to evaluate the benefit of an intervention; thus, the available literature on the quality of life of CI patients is very heterogenous.

Therefore, the aim of this study was to conduct a systematic literature review on the generic QoL of CI users, as well as to provide additional audiological data and QoL scores from a pediatric study cohort of CI users from Romania. Therefore, the following points were the focus of this paper:

- A systematic review of the current literature regarding the QoL of CI users was conducted to obtain an overview of the generic QoL questionnaires utilized in the literature, and to see which questionnaires were employed for pediatric CI users.
- The generic QoL of the Romanian CI user study cohort was assessed, and they were compared with age- and gender-matched peers with normal hearing.
- Data from the study cohort were collected to assess the level of speech comprehension and hearing ability of pediatric CI users in Romania using the translated and recorded HSM sentence test and pure tone audiometry.
- Possible influencing factors, such as early-implanted (≤3 years) CI users versus late-implanted (>3 years) children, were investigated.
- Finally, possible correlations between the generic and health-related quality of life questionnaires were assessed.

## 2. Materials and Methods

The methods can be divided into two main areas, with one area describing the methods section for the systematic review. The second main item contains the data collection methods for the Romanian study group of pediatric CI users.

### 2.1. Systematic Review

The systematic review was conducted according to the PRISMA guidelines [12]. The studies were searched in the PubMed database according to the search terms defined in Table 1. Inclusion and exclusion criteria were defined based on PRISMA guidelines [12] using the so-called PICOS classification (Population, Intervention, Comparators, Outcomes, and Study Design), and are listed in Table 2. The systematic review was not registered.

The Oxford level of evidence guidelines were considered in order to evaluate the quality of the included papers [13]. The five levels were defined based on the possible outcomes/research standards, with level I representing the best and level V representing the worst reporting standard. In our analysis, for example, we considered the study design, the number of study participants, the follow-up examinations, etc.

### 2.2. Data Collection from the Study Cohort in Romania

#### 2.2.1. Participants

For this study, 93 participants, with a mean age of 10.9 ± 5.0 years, were included. The study cohort consisted of 35 female and 58 male children and adolescents from Romania who were suffering from sensorineural hearing loss accompanied with a hearing threshold higher than 70 dB. Every participant was fitted unilaterally with a cochlear implant (Combi 40+, Pulsar, Sonata, Mi1000 Concerto, Mi1200 Synchrony) and an audio processor (behind the ear: Opus, Opus 2, Sonnet, Sonnet 2, Tempo+; single unit: Rondo, Rondo 2, Rondo 3) from MED-EL (MED-EL GesmbH, Innsbruck, Austria). Adolescents and adults up to 20 years of age were included if the implantation had been carried out in childhood. For this reason, the age range extended from 2 to 20 years.

#### 2.2.2. Data Collection

Data were collected during clinical routine measurements at the clinic of Timisoara (Municipal Hospital, Strada Gheorghe Dima nr. 5, Timisoara, Romania), as well as from a CI self-help group camp in Eforie. The relevant patient outcomes gathered by responsive clinical personnel were provided in a pseudonymized form. The parents or legal representatives provided their consent for the collection of their children’s data.

#### 2.2.3. Quality of Life Assessments

The QoL questionnaires were completed by the participants themselves, using either the online form or the printed version. If the study participants were too young, the questionnaires were completed with the help of their parents or legal representatives.

##### Assessment of Quality of Life (AQoL-6D)

To measure the generic QoL, the AQoL-6D total utility score, along with the individual utility scores of the six dimensions, were assessed. These dimensions were as follows: Independent Living, Mental Health, Coping, Relationships, Pain, and Senses [14]. The AQoL with six dimensions was considered because the participants were children and adolescents, and the questionnaire with eight dimensions is designed for adults only [15]. The AQoL-6D consists of 20 items, each with four to six possible answers to describe the subject’s situation [14]. Each answer option is assigned a numerical value from 1–6, depending on how many answers are available, and the evaluation tools provided by the AQoL-Homepage convert the absolute outcomes into weighted utility scores [14,16]. In general, the total utility score and the utility sub-scores of the six dimensions for the AQoL-6D range from 0 to 1, with 0 being the worst and 1 being the best possible result, indicating a perfect QoL [14].

##### Speech, Spatial and Qualities of Hearing Scale (SSQ12)

The SSQ12 consists of twelve questions separated into three main dimensions and an overall outcome [6]. Each question is answered using a Likert scale from 0 to 10. The average of each category (speech, spatial, quality of hearing, and the overall average) is used for the outcome calculations. The resulting outcomes range from 0 to 10, with 10 being the best possible result and 0 the worst. Thus, a score of 10 represents no limitations on QoL due to hearing impairment [17].

#### 2.2.4. Audiological Measurements

##### Sound Field Audiometry (Clinical Set-Up)

Sound field measurements are frequently performed in routine audiological examinations to determine the hearing thresholds over several frequencies [18]. Calibrated loudspeakers (Pioneer Corp., S-SP 50, Tokyo, Japan) are placed in front of the participant, and sounds of different volumes and frequencies are presented via an audiometer (Interacoustics A/S, AA222, Middelfart, Denmark) in a soundproof room. The non-implanted side is plugged and covered to determine the hearing level of the implanted ear [19].

##### Speech Audiometry (Camp Set-Up)

The Hochmaier–Schulz–Moser (HSM) sentence test was the assessment of choice for this study where recited sentences must be repeated by the attendant [20]. It was specifically developed to assess the speech comprehension of CI users, and consists of 30 lists with 20 daily sense sentences for each test set. Each list has a word count of 106 words [9]. The HSM sentence test was translated into Romanian and recorded by a professional native male speaker. The results ranged from 0% to 100%, with 100% representing a perfect intelligibility of speech.

#### 2.2.5. Data Analysis

The primary AQoL-6D analysis was conducted with the evaluation tool provided by the AQoL Homepage [14] for the statistical software SPSS (IBM Corp.; IBM SPSS Statistics 29; Armonk, NY, USA). Statistical analysis was carried out using the GraphPad PRISM statistical software (GraphPad Software Inc.; demo version or latest version 9.0.0; San Diego, CA, USA).

The pure tone averages (PTAs) for hearing thresholds with frequencies of 0.5, 1, 2, and 4 kHz were calculated [19,21] for further comparative statistical analyses.

All data sets were tested for normal distribution by applying the Shapiro–Wilk test with a confidence level of 95%, resulting in no normal distribution. Nonparametric statistical tests like the Mann–Whitney U-test, the Spearman rank correlation test, and the Dunn’s multiple comparison were also used. A significance level with an alpha value of 0.05 was set for all tests.

## 3. Results

There are two primary sections in the results section. The first describes the systematic review’s outcomes. The results of the Romanian study group of pediatric CI users are included in the second main part. Furthermore, unless otherwise specified, all results and numerical values are presented as the mean and standard deviation (SD).

### 3.1. Systematic Review

The presented outcomes of this systematic review include the results of the screening process; the investigation of the quality of life questionnaires for adults and children that are represented in the current literature; and, finally, the evaluation of the extracted studies.

#### 3.1.1. Screening Process

**Figure 1:**
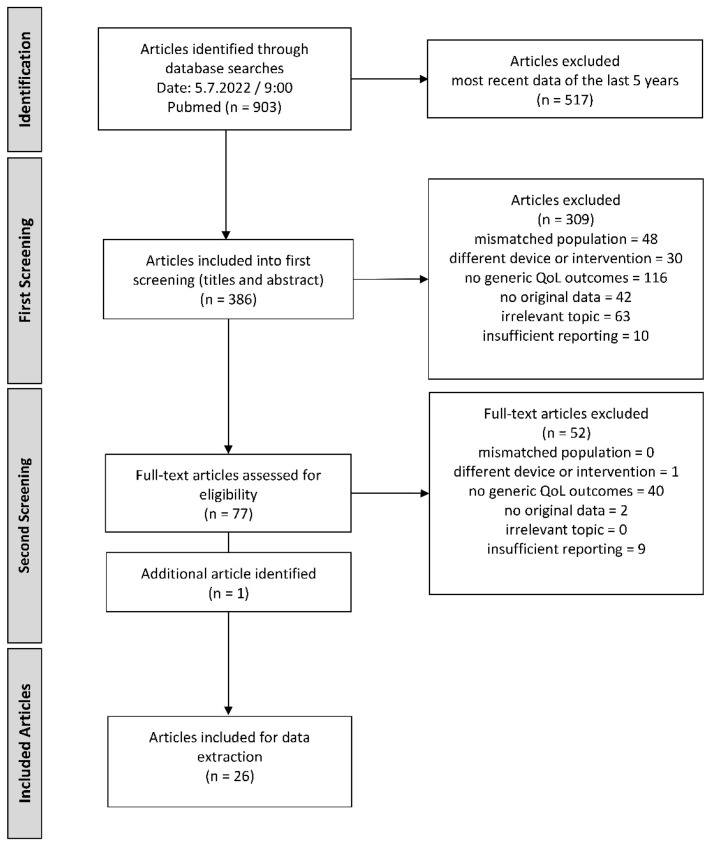
Flow diagram of the study selection process according to the PRISMA guidelines [12] (search conducted on 05.07.2022). QoL = quality of life.

The process and outcomes of the systematic literature search can be seen in the flow chart in Figure 1. First, the articles were selected according to the title and abstract, leaving 77 papers for the second screening, which involved the full text. Following the second screening, data extraction was performed for 25 publications. The screening outcomes and reasons for exclusion (*n* = 157) are outlined in Figure 1.

An additional search, specifically for the AQoL with the search terms (cochlear implant *) AND (aqol), was conducted on 28.7.2022/9:00. Two publications were identified, but only one [15] met the inclusion criteria described in Table 2. Finally, 26 publications were included for further data extraction.

#### 3.1.2. Adult CI Users and Their Generic QoL

The extracted data of the generic QoL scores for adult CI users are presented in Table 3. The darker blue fields emphasize the total scores, while the lighter green and blue fields highlight the subcategories that can also be merged. An example of this can be seen for the AQoL at the bottom of Table 3, where the light blue subcategories of independent living, pain, and senses are combined to form the category of physical super dimension. The given standard deviation was calculated for the summarized mean values of the included publications. If only one publication is provided for a number of papers, it indicates that the values were extracted directly from the published article. As shown in Table 3, the quantity of the study cohort mostly varied according to the number of subjects from whom data were collected. The reason for this was that several questionnaires were administered, but did not always include the entire group. In the follow-up investigations, it also happened that patients were lost to follow-up for unknown reasons.

In general, it can be seen in Table 3 that the QoL was nearly always higher for all scores after a cochlear implant intervention. However, the dimensions and numerical ranks differed so much that the questionnaires could not be compared with each other.

#### 3.1.3. Pediatric CI Users and Their Generic QoL

Table 4 shows the generic QoL outcomes for children and adolescents. The systematic review included three QoL questionnaires for children. For the children and adolescents, only postoperative outcomes were recorded. As with the adult population, a higher outcome of the questionnaire generally indicates a better quality of life. It was visible that there is no uniform way to measure quality of life in children. In general fewer data were available for children.

#### 3.1.4. Quality Evaluation of Extracted Publications

**Figure 2:**
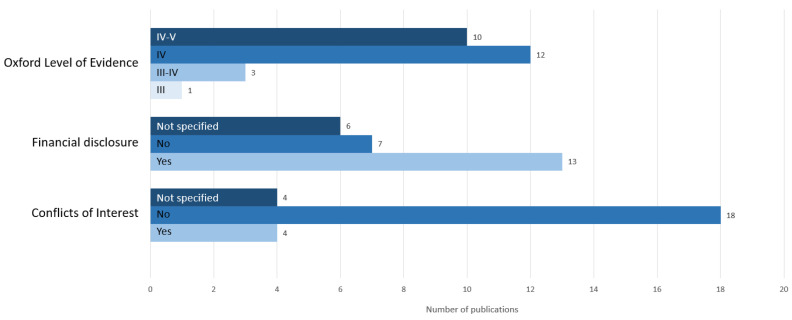
Quality evaluation of the included publications of the systematic review according to the Oxford level of evidence [13]. Additional representation of financial support and possible conflicts of interest.

To evaluate the quality of the included papers, the Oxford level of evidence was used, which ranges from level I to V, with the best rating corresponding to level I. Most papers indicated only level IV or IV–V, because they were case series or case–control studies, which can be seen in Figure 2. The prospective cross-sectional study by Issing et al. [35] achieved level III because long-term follow up evaluations with more than 30 participants, as well as comparison group data, were provided, and the outcomes were presented clearly and understandably to the reader. In addition, we investigated whether the studies had been financially supported, which was the case for 13 publications out of 26. It can also be seen in the bar chart in Figure 2 that 18 papers did not report conflicts of interest, only four did, and four did not provide this information.

### 3.2. Data Collection from Study Cohort in Romania

#### 3.2.1. Assessment of Quality of Life (AQoL-6D)

**Figure 3:**
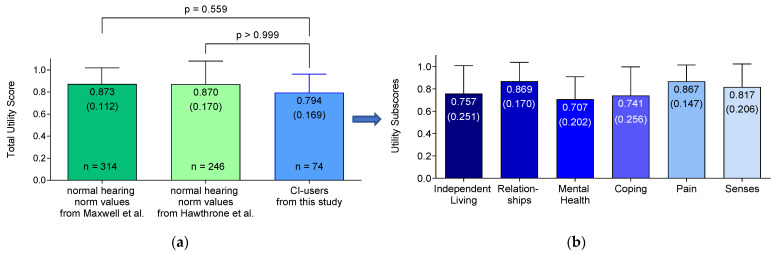
Graphical representation of the total utility score ((**a**), blue bar) and the utility sub-scores (**b**) on the assessment of quality of life questionnaire, which included six dimensions (AQoL-6D), for cochlear implant (CI)treated children (*n* = 74) of this study An additional comparison with the total utility scores on the AQoL-6D using norm values of peers of the same age and gender with normal hearing is also shown, obtained from the studies of Maxwell et al. [43] ((**a**), dark green) and Hawthorne et al. [44] ((**a**), light green). Significance level: *p* < 0.05. The mean values and corresponding standard deviations in parentheses are displayed in the bars. The scores range from the worst score of 0, indicating no quality of life (QoL), to the maximum score of 1, indicating a perfect generic QoL.

Figure 3 represents the results of the total utility score and utility sub-scores of the AQol-6D questionnaire from the pediatric CI users of this study in blue. It is important to note that the blue total utility score in the left chart of Figure 3a is composed of the six dimensions of the right bar chart (b), which provides the utility sub-scores. In general, a utility score of 0 indicates no QoL, while a maximum score of 1 represents a perfect QoL. The results of the individual dimensions of the 74 participants in this study ranged in utility score from 0.71 to 0.87. In Figure 3, the highest scores were achieved in the relationships (0.87 (0.17)) and pain (0.87 (0.15)) dimensions. However, the participants had the lowest results for the sub-scores of mental health (0.71 (0.20)) and coping (0.74 (0.26)).

The total utility score of the AQoL-6D of this study was 0.79 (0.17) for the children and adolescents who had been treated with a cochlear implant, which can be seen in the blue bar in the left bar chart of Figure 3a. Furthermore, in the left bar chart in Figure 3a, norm values for the total utility scores of age- and gender-matched peers with normal hearing are presented in green. These norm values were extracted from the publication by Maxwell et al. [43] (represented as a dark green bar), as well as from the study by Hawthorne et al. [44] (represented as a light green bar). Both values from the indicated publications [43,44] were compared to the total utility score of the AQoL-6D of this study. Neither the norm values of Hawthorne et al. [44] (*p* = > 0.999) nor those from Maxwell et al. [43] (*p* = 0.559) were able to demonstrate a significant difference (*p* < 0.05) between the total utility score of CI users and children with normal hearing.

#### 3.2.2. Speech Spatial Qualities of Hearing Scale (SSQ12)

**Figure 4:**
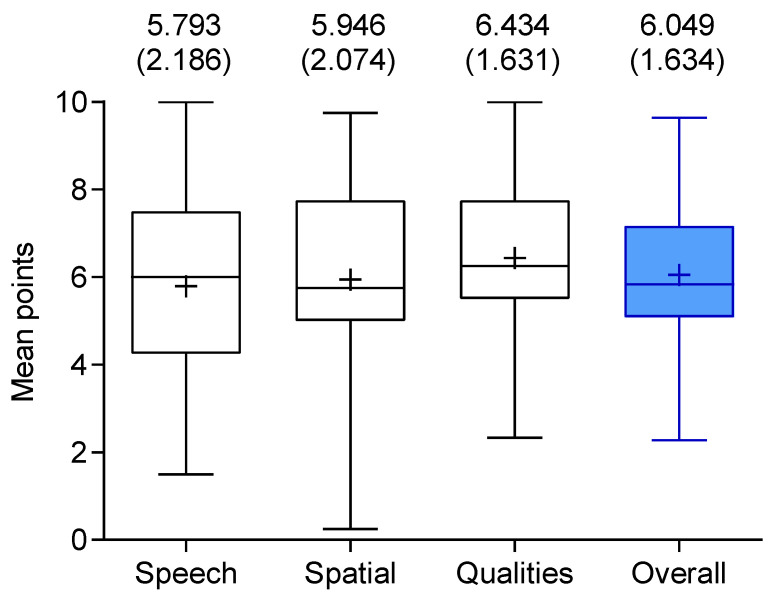
Boxplot presentation of the Speech Spatial Qualities of Hearing Scale questionnaire (SSQ12) outcomes from 79 cochlear implant users. Median and quartiles for the sub-scores and the overall score (blue) are presented. An additional representation of the mean values are shown as crosses inside the boxplots and as numerical values above the boxplots, with their standard deviations in parentheses. The score, in general, ranges from a worst possible score of 0 to an optimal score of 10. A maximum score of 10 represents no limitations on quality of life due to hearing impairment.

The results of the SSQ12 questionnaire for 79 pediatric CI users are shown in Figure 4. The individual scores on the speech spatial qualities of hearing test are displayed. The total score is presented in blue. The results ranged from 0 to 10, with 0 corresponding to the worst result and 10 to the best. A maximum score of 10 points would indicate that there is no limitation to quality of life due to the hearing impairment. In general, the mean values in Figure 4 are represented as crosses, and the mean value of the overall score of the SSQ12 for CI users is 6.05 (1.63). The highest score of 6.43 (1.63) was achieved in the qualities of hearing section. Hence, the lowest score, 5.79 (2.19), was given for the speech category.

#### 3.2.3. Pure Tone Audiometry

**Figure 5:**
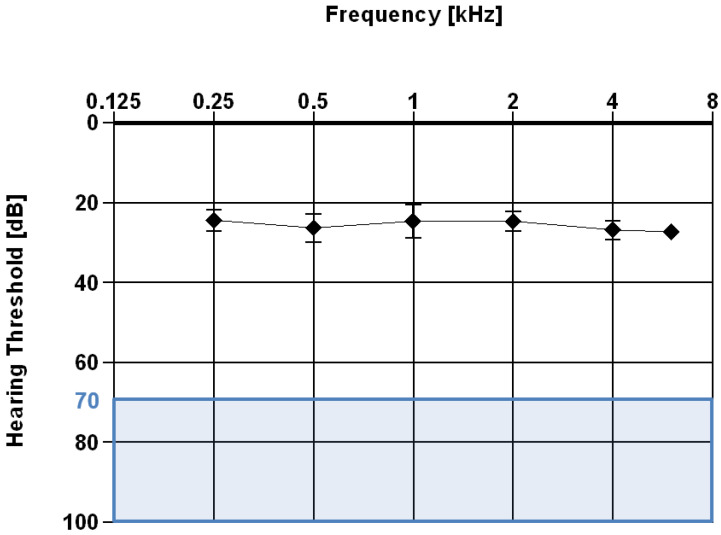
Audiogram with averaged hearing thresholds and standard deviations of the children and adolescents with cochlear implants (CI). For each frequency, data from at least 40 CI users were considered, but the exact numbers of participants are noted in Table 5. The blue area shows the CI indication range based on the manufacturer’s recommendation [45].

Pure tone audiometry refers to the total number of examined ears which were treated with a cochlear implant (Table 5). The best aided hearing level was 24.4 dB, measured at a frequency of 0.25 kHz, as shown in (Figure 5, Table 5). The hearing threshold ranged from 24.4 dB (2.62 dB), at a test frequency of 0.25 kHz, to 27.3 dB (1.52 dB) at a frequency of 6 kHz.

The blue area at the bottom of the audiogram represents the cochlear implant indication range of the manufacturer (MED-EL [45]) (Figure 5). The hearing thresholds of most CI users improved by at least 40 dB after implantation.

#### 3.2.4. Speech Audiometry

The HSM sentence test was conducted at a self-help camp in Romania, and 41 children and adolescents with cochlear implants successfully completed the test which can be seen in Table 6. Overall, a mean score of 71.6% (23.8) was achieved by the young CI users.

#### 3.2.5. Implantation Timepoint

The study cohort was split into early (≤3 years) and late (>3 years) implantation groups. The assessed quality of life (AQoL-6D and SSQ12) and audiological (PTA and HSM sentence test) parameters were compared with each other. No significant differences between the early and late implantation groups were found for any of the four comparative parameters (Table 7).

#### 3.2.6. Generic and Health-Related Quality of Life

A moderate and significant correlation between the generic (AQoL-6D) and the health-specific (SSQ12) questionnaires was found, with a Spearman coefficient of 0.4370 [47]. Additionally, the relationship between the utility score of the senses dimension of the AQoL-6D compared to the SSQ12 was investigated. With a Spearman coefficient of 0.6739, a moderate and significant correlation was found (Table 8).

## 4. Discussion

From the generic QoL results, it can be concluded that CI users can achieve the same level of quality of life as their healthy, normal-hearing peers. After comparing the normative values from two different publication populations [43,44], no significant difference regarding the QoL was detected between normal-hearing and CI-treated children and adolescents (*p* < 0.05). Although the absolute numerical norm values achieved by Hawthorne et al. [44] (0.870 (0.170)) and Maxwell et al. [43] (0.873 (0.112)) were somewhat different, a multiple comparison method revealed no significant difference in utility scores compared to the outcomes of this study (0.794 (0.169)), supporting the statement that CIs increase the QoL of hearing-impaired people after treatment [4] and confirming that normal hearing standards can be achieved. A possible influence of the fact that some questionnaires were answered by or together with the parents due to the age of the child and more heterogenous outcomes, as reflected by a rather high standard deviation (SD) such as that reported by the study of Khadka et al., could not be seen in our results, which achieved a rather low SD of 0.169 [48]. The validated norm values were retrieved from age groups of 15–19 years in the publication by Hawthorne et al. [44] and 16–24 years in the study of Maxwell et al. [43].

However, the overall positive outcomes are probably the result of an increase in self-confidence bolstered by the ability to communicate [6]. As no statistical gender differences could be found, the outcomes were not split (*p* > 0.05).

A total of 79 participants achieved a mean overall score of 6.05(1.63) for the health/hearing-related QoL, assessed by the Speech Spatial Qualities of Hearing Scale (SSQ12), which appears to be rather low in comparison to other publications [49,50,51,52]. Emphasis needs to be placed on the rather young study cohort, in which speech might not have been as developed due to age, not due to hearing status. It is also possible that the comparative studies simply presented the results of small, top-performing groups. For example, the studies by Rauch et al. [49] and Falcon et al. [50] involved single-sided deaf patients with normal hearing on one side [53].

A moderate and significant correlation between the outcomes of the generic and health/hearing-specific QoL was detected (Table 8). Unsurprising, the AQoL-6D subdimension of senses, which deals with seeing, hearing, and communication, showed the best correlation with the SSQ12, again reflecting the importance of hearing on overall quality of life. Parameters like anxiety, feelings of seclusion, or depression, which very often accompany untreated hearing loss [2], are often evaluated, and may be eliminated via treatment.

Even though the literature on AQoL assessments in the population with hearing loss is sparse, the questionnaire was used as the assessment of choice for utilization in the clinical process. The reasons were as follows: first, access was free of charge; second, the statistical software SPSS and STATA provided easy and, therefore, bias-free evaluation tools; and third, the availability of validated population norms enabled statistical comparisons. The provided data calculation syntax for STATA and SPSS ensures correct data analysis and, therefore, better comparability. Thus, no random deviations or obvious calculation errors occurred, as was found in the published outcomes of several HUI 3 questionnaire studies. The HUI was often evaluated incorrectly, which unfortunately made the results unusable for our systematic review and comparisons, especially since a license fee was required to for this questionnaire.

During the review process of the different QoL studies, it became evident that a consensus on the application of QoL instruments would be advisable.

Unfortunately, not enough comparable data were available to perform a meta-analysis (Table 3 and Table 4) [1,15,16,22,23,24,25,26,27,28,29,30,31,32,33,34,35,36,37,38,39,40,41,42,54,55]. Although no age group was excluded from the literature review, it is apparent that there is limited data available regarding QoL surveys on children [1,38,39,40,41,42]. This was one of the reasons why this study focused on collecting data from children and adolescents.

In conclusion, the systematic literature review clearly showed improved QoL after implantation, comparable to that of healthy, normal-hearing children, but also showed some clear limitations: first, a clear classification of health-related and generic QoL questionnaires is necessary. Second, uniform and correct assessments are needed in order for comparisons to be made. Third, more day-to-day evidence is required, which points towards the necessity to implement QoL questionnaires into clinical routines. More data on the QoL of the pediatric population are especially required.

The 41 tested CI users achieved impressive speech comprehension, with an overall score of 71.6% (23.8%) on the HSM sentence test (Table 6). The rather high standard deviation might be because of overly complex sentences or words, even though the HSM test includes everyday phrases in order to eliminate this possible bias. Another possibility might be the concentration level or the unfamiliar environment, or that the children were simply too shy to repeat the words [56].

The tested CI users showed an improvement of around 40 dB with the use of a cochlear implant, as the mean aided hearing threshold was between 24.4 dB (2.62 dB) and 27.3 dB (1.52 dB).

The literature repeatedly points out the importance of early implantation to ensure the optimal developmental effects [57,58,59]. Our study group outcomes were split into early- and late-implanted children (Table 7). Children under 3 years of age at the time of implantation were considered to fall into the early implantation group, and the rest comprised the late implantation group. Despite the outcomes not being significantly different, the early implantation group performed about 10% better than the comparison group on the HSM sentence test.

In conclusion, both age groups achieved generally good results for all measurements which were undertaken. These great results are probably related to the support of the families and their social environments. This assumption is based on the fact that all examinations, as well as rehabilitation and training (in the camp as well as in the hospital in Timisoara), were voluntary, and the parents went above and beyond to provide their children with all the opportunities necessary to further improve their hearing. Obviously, they were doing everything right.

## 5. Conclusions

Studies investigating the effect of a CI on different aspects of QOL and audiologic performance are important in order to provide realistic expectations to our patients, especially when children are involved. This study has proven that cochlear implant users can achieve the same generic quality of life as their normal-hearing peers. The audiological examinations showed a clear benefit after cochlear implantation. Since speech comprehension is challenging, the good outcomes achieved on the HSM sentence test by the CI users in Romania were impressive. Even though no significant difference was found between late and early implantation, for the QoL and audiological parameters examined, the early implantation group exhibited roughly 10% greater speech understanding compared to the later implantation cohort. No significant correlation between the generic and health-related quality of life questionnaires was found. Thus, both should be administered to obtain a better understanding of children and adolescents treated with cochlear implants and their hearing/health, as well as their general satisfaction in life.

## Figures and Tables

**Table 1:** Search terms and hits for the literature search in the PubMed database.

Search Steps	Search Terms	Hits
1	((Quality of Life) AND (cochlea * implant *))	903
2	Limit NOT (Health Related Quality of Life)	903
3	Filter: last five years for recent data (from 05/07/2017 to 05/07/2022)	386

**Table 2:** Inclusion and exclusion criteria for literature screening.

**Inclusion Criteria**	
Population	Subjects of any age, gender, or ethnicity with any hearing loss or single-sided deafness (SSD), as well as and their legal representatives
Intervention/treatment	Cochlear implantation
Comparator	Not applicable
Outcomes	Generic quality of life scores
Study design	All studies with original data, including case series, case–control, and longitudinal studies. *Systematic reviews without original data, as well as acknowledgements, recommendations, or general topic descriptions were excluded*.
**Exclusion Criteria**	
	Different device or treatment Not a clinical study in humans Other type of hearing loss (not SNHL, MHL, or SSD) Topic not related to hearing loss or its treatment Publication lacking sufficient information for evaluation Overlap of data

**Table 3:** Extracted data regarding the systematic review for adults. All questionnaires refer to the generic quality of life of cochlear implant users before and after the intervention. If more than one publication is indicated, the values correspond to the mean value of these studies and their standard deviation (SD), unless otherwise stated. If medians and ranges are specified, they are listed separately. Darker blue fields represent total scores and lighter green or blue fields highlight the subcategories of the SF-36.

Range	Category	Evaluation Time	Mean Score	SD	Median	Range	n Specific Test for		Age Mean [years]	Age SD [years]	n Whole Study Cohort	n Publications Included	Publications
							Mean Values	Median Values					
**1. GBI (=Glasgow Benefit Inventory)**													
**−100 to +100**	Total	Postoperative	39.6	9.99	-	-	240	-	56.4	11.6	280	6	Anzivino et al., 2019 [22] Calvino et al., 2022 [23] Hey et al., 2019 [24] Sivonen et al., 2021 [25] Sorrentino et al., 2020 [26] Tang et al., 2017 [27]
	General		51.19	14.40	25	10.8–33.33	322	27	57.0	11.2	391	7	Calvino et al., 2022 [23] Forli et al., 2019 [28] Hey et al., 2019 [24] Peters et al., 2021 [29] Sivonen et al., 2021 [25] Sorrentino et al., 2020 [26] Tang et al., 2017 [27]
	Social		28.65	11.86	0	0–8.33							
	Physical		13.70	17.79	0	0–0							
	Total	postoperative short term (<12 months)	38.00	9.80	-	-	67		63.6	2.9	70	2	Anzivino et al., 2019 [22] Hey et al., 2019 [24]
	General		38.80	27.70	25	10.8–33.33	42	27	57.0	3.8	74	2	Hey et al., 2019 [24] Peters et al., 2021 [29]
	Social		10.50	29.70	0	0–8.33							
	Physical		3.20	15.70	0	0–0							
	Total	postoperative long term (≥12 months)	40.06	10.00	-	-	173	-	54.0	12.4	210	4	Calvino et al., 2022 [23] Sivonen et al., 2021 [25] Sorrentino et al., 2020 [26] Tang et al., 2017 [27]
	General		52.43	14.54			280		57.0	12.3	317	5	Calvino et al., 2022 [23] Forli et al., 2019 [28] Sivonen et al., 2021 [25] Sorrentino et al., 2020 [26] Tang et al., 2017 [27]
	Social		30.46	10.88									
	Physical		14.75	18.33									
**2. WHOQOL (=World Health Organisation, Quality of Life)**													
**WHOQOL-BREF (short form)**													
0–100	Total	Postoperative	66.00	11.75	-	-	97	-	44.4	3.6	97	4	Chen et al., 2022 [30] Peter et al., 2019 [31] Saraç et al., 2019 [32] Sousa et al., 2018 [33]
	Environment		59.70	4.60									
	Social realitionships		59.17	7.93									
	Physical health		63.50	6.82									
	Psychosocial health		61.40	10.09									
**WHOQOL-OLD (for old age)**													
0–100	Total	Preoperative	60.00	15.70	-	-	34	-	73.5	4.9	34	1	Issing et al., 2020 [34]
	Sensory Abilities		38.10	22.60									
	Autonomy		63.20	17.60									
	Past, Present, Future Activities		66.20	18.00									
	Social Participation		61.40	21.00									
	Death and Dying		61.90	30.00									
	Intimacy		69.30	20.20									
	Total	Postoperative	69.04	3.41	-	-	151	-	71.5	4.1	178	3	Issing et al., 2020 [34] Issing et al., 2022 [35] Völter et al., 2018 [36]
	Sensory Abilities		55.18	1.65	-	-	118	-	74.4	0.9	118	2	Issing et al., 2020 [34] Issing et al., 2022 [35]
	Autonomy		69.80	3.36									
	Past, Present, Future Activities		71.63	3.00									
	Social Participation		68.90	2.49									
	Death and Dying		65.53	5.69									
	Intimacy		73.73	1.39									
**3. SF-36 (=Short Form Health Survey with 36 items)**													
0–100	Physical health	Preoperative	61.51	13.21	-	-	55	-	56.2	10.2	55	2	Anzivino et al., 2019 [22] Chen et al., 2022 [30]
	Mental health		55.05	2.06									
	Physical Functioning		-	-	57.20	55.1–57.2	-	30	median: 35	range: 16–54	30	1	Forli et al., 2017 [37]
	Role-Physical				56.20	49.2–56.2							
	Body Pain				62.80	47.3–62.8							
	General Health				57.90	50.9–61.7							
	Vitality				53.80	46.7–60.9							
	Social Functioning				46.30	35.4–57.2							
	Role-Emotional				55.30	44.8–55.3							
	Mental Health				45.90	39.1–55.0							
	Physical health	Postoperative	69.20	9.30	-	-	55	-	56.2	10.2	55	2	Anzivino et al., 2019 [22] Chen et al., 2022 [30]
	Mental health		66.43	5.37									
	Physical Functioning		-	-	57.20	55.1–57.2	-	30	median: 35	range: 16–54	30	1	Forli et al., 2017 [37]
	Role-Physical				56.20	49.2–56.2							
	Body Pain				62.80	51.6–62.8							
	General Health				60.30	54.6–61.7							
	Vitality				56.20	51.1–63.3							
	Social Functioning				49.00	40.9–57.1							
	Role-Emotional				55.30	55.3–55.3							
	Mental Health				52.70	45.9–55.0							
**4. GHSI (=Glasgow Health Status Inventory)**													
−100 to +100	Total	postoperative	66.00	2.00			23		44.2	range: 19.5–64.8	27	1	Sivonen et al., 2021 [25]
**5. EQ-5D-5L (=European Quality of Life in 5 Dimensions, 5 Level Version)**													
0–5	Mobility	postoperative shortterm (<12 months)	1.65	0.95	-	-	20	-	3.0	5.8	458	1	Piromchai et al., 2021 [38]
	Self-care		1.15	0.67									
	Usual activities		1.45	0.83									
	Pain/discomfort		1.55	0.60									
	Anxiety/depression		1.65	0.99									
	Mobility	postoperative longterm (≥12 months)	1.29	0.59	-	-	17						
	Self-care		1.12	0.33									
	Usual activities		1.47	0.72									
	Pain/discomfort		1.47	0.51									
	Anxiety/depression		1.18	0.39									
**6. HUI 3 (=Health Utilities Index)**													
0–1	Total	preoperative	0.56	-	-	-	59	-	72.3	6.8	59	1	Sarant et al., 2019 [16]
		postoperative	0.67	-			20						
**7. AQoL-8D (=Assessment of Quality of Life in 8 Dimensions)**													
0–1	Total	Preoperative	0.50	0.23	-	0.17–0.99	104	-	34.8	16.6	104	1	Rostkowska et al., 2021 [15]
	Physical superdimension		0.50	0.20		0.15–0.91							
	Psycho-social superdimension		0.27	0.19		0.05–0.92							
	Independent living		0.78	0.15		0.39–1							
	Pain		0.76	0.24		0.15–1							
	Senses		0.53	0.18		0.25–0.97							
	Mental health		0.53	0.16		0.25–1							
	Happiness		0.64	0.18		0.24–1							
	Coping		0.72	0.17		0.38–1							
	Relationships		0.60	0.14		0.47–1							
	Self-worth		0.64	0.21		0.28–1							
	Total	Postoperative	0.66	0.19	-	0.21–0.99							
	Physical superdimension		0.62	0.20		0.18–0.96							
	Psycho-social superdimension		0.37	0.18		0.09–1							
	Independent living		0.86	0.14		0.35–1							
	Pain		0.78	0.22		0.21–1							
	Senses		0.71	0.16		0.35–0.97							
	Mental health		0.60	0.13		0.29–1							
	Happiness		0.73	0.13		0.36–1							
	Coping		0.80	0.12		0.52–1							
	Relationships		0.70	0.14		0.47–1							
	Self-worth		0.81	0.14		0.39–1							

**Table 4:** Extracted data regarding the systematic review for children. All questionnaires refer to the generic quality of life of cochlear implant users after the intervention. If more than one publication is indicated, the values correspond to the mean value of these studies and their standard deviation (SD). If medians and ranges are specified, they are listed separately. Darker blue fields represent total scores.

Range	Category	Evaluation Time	Mean Score	SD	Median	Range	n Specific Test for		Age Mean [years]	Age SD [years]	n Whole Study Cohort	n Publications Included	Publications
							Mean Values	Median Values					
**8. PedsQL (=Pediatric Quality of Life Inventory)**													
**0–100**	**Physical**	**postoperative shortterm** **(<12 months)**	**74.59**	**23.67**			**23**		**2.96**	5.83	458	1	Piromchai et al., 2021 [38]
	Emotional		56.96	18.63									
	Social		50.22	22.94									
	School		50.94	32.08									
	Total	postoperative longterm (≥12 months)	87.08	11.10	79.31	34.78–100	73	34	4.79	1.22	107	2	Alnuhayer et al., 2020 [1] Hendriksma et al., 2020 [39]
	Physical		90.04	2.93	89.36	40.63–100	81	34	3.88	0.92	566	3	Alnuhayer et al., 2020 [1] Hendriksma et al., 2020 [39] Piromchai et al., 2021 [38]
	Emotional		72.07	15.19	73.33	25–100							
	Social		75.23	12.10	78.24	10–100							
	School		66.20	14.53	76.50	37.5–100							
**9. GCBI (=Glasgow Children’s Benefit Inventory)**													
−100 to +100	Total	postoperative	58.12	5.89			127		3.54	0.24	127	2	Aldriweesh et al., 2021 [40] Dev et al., 2022 [41]
	Physical health		44.30	14.10			57		3.30	1.9	57	1	Dev et al., 2022 [41]
	Emotion		68.10	13.20									
	Learning		78.90	10.70									
	Vitality		64.70	12.10									
**10. KINDL**													
0–100	Total	postoperative	46.90	12.00			34		5.33	0.98	34	1	Vermi Sli Peker et al., 2020 [42]
	Physical well-being		67.70	16.20									
	Self-esteem		42.70	22.70									
	School/Kindergarten		41.20	22.10									
	Social Relations (Friends)		45.40	17.90									
	Emotional Well-Being		41.00	17.10									
	Family		44.90	19.10									

**Table 5:** Mean values of the hearing thresholds and their standard deviations (SDs) at the specific test frequencies of the pure tone audiometry for the cochlear implant users of this study. The degrees of hearing loss (HL) were determined by the guidelines of American Speech-Language-Hearing-Association (ASHA) [46].

Frequency (kHz)	Hearing Threshold, Mean (dB)	SD	*n* (Ears)	Degree of Hearing Loss according to ASHA
0.25	24.4	2.62	55	Slight HL
0.5	26.3	3.47	56	Mild HL
1	24.6	4.06	55	Slight HL
2	24.6	2.34	55	Slight HL
4	26.8	2.21	51	Mild HL
6	27.3	1.52	45	Mild HL

**Table 6:** Results of the Hochmaier–Schulz–Moser (HSM) sentence test for 41 cochlear implant users. The scores generally ranged from a minimum score of 0% to a maximum score of 100%, indicating perfect speech comprehension.

Properties	Scores
Participants (*n*)	41
Mean (%)	71.6
Standard deviation (%)	23.8
Minimum (%)	15.1
Maximum (%)	100

**Table 7:** Comparative values between early (≤3 years) and late (>3 years) implantation timepoints for cochlear implant users.

Comparison Parameters	Early Implantation ≤3 Years	*n*	Late Implantation >3 Years	*n*	*p*-Value	Significantly Different (*p* < 0.05)
AQoL-6D	0.78 (0.19)	37	0.81 (0.14)	37	0.7252	No
SSQ12	5.97 (1.79)	41	6.13 (1.47)	38	0.5995	No
PTA	25.6 dB (12.8 dB)	27	21.2 dB (7.01 dB)	20	0.1127	No
HSM sentence test	72.9% (24.5%)	22	70.1% (23.5%)	19	0.6283	No

The following parameters were compared: Assessment of Quality of Life in six dimensions (AQoL-6D), Speech Spatial Qualities of Hearing Scale (SSQ12), Pure Tone Average (PTA), and the Hochmair–Schulz–Moser (HSM) sentence test. The comparison parameters are significantly different when the *p*-value is <0.05.

**Table 8:** Correlation characteristics of the total scores of the generic (Assessment of Quality of Life in six dimensions (AQoL-6D)) and health-related (Speech Spatial Qualities of Hearing Scale (SSQ12)) quality of life. The correlation is significant when the *p*-value is <0.05.

Correlation Parameter	*n* Pairs	Spearman ρ	Correlation Strength	*p*-Value	Correlation Significant (*p* < 0.05)
AQoL-6D and SSQ12	72	0.4370	moderate	0.0001	Yes
AQoL-6D Senses and SSQ12	72	0.6739	moderate	<0.0001	Yes
